# Achievement and Enhancement

**DOI:** 10.1017/can.2019.43

**Published:** 2020-04

**Authors:** Lisa Forsberg, Anthony Skelton

**Affiliations:** 1 Faculty of Law, Oxford Uehiro Centre for Practical Ethics, and Somerville College, University of Oxford, Oxford, United Kingdom; 2 Department of Philosophy, University of Western Ontario, London, Canada

**Keywords:** Achievement, difficulty, effort, competent causation, well-being, perfectionism, enhancement

## Abstract

We engage with the nature and the value of achievement through a critical examination of an argument according to which biomedical “enhancement” of our capacities is impermissible because enhancing ourselves in this way would threaten our achievements. We call this the argument against enhancement from achievement. We assess three versions of it, each admitting to a strong or a weak reading. We argue that strong readings fail, and that weak readings, while in some cases successful in showing that enhancement interferes with the nature or value of achievement, fail to establish that enhancement poses an unusual threat to achievement.

Achievement, at one time neglected, is now a growing preoccupation in normative ethics. In this paper, we engage with the nature and the value of achievement through a critical examination of the argument that “enhancement”^1^ of our capacities is impermissible because it would threaten our achievements. We call this the argument against enhancement from achievement, or the AEA. We construct the argument and determine its strength and its effect on the philosophical understanding of achievement’s nature and its value.

We articulate three versions of the AEA; each admits to a strong or a weak reading. We argue that strong readings fail to establish that enhancement always interferes with the nature or value of achievement and that weak readings, while in some cases successful in showing that enhancement interferes with the nature or value of achievement, fail to establish that enhancement poses an unusual threat to achievement. Our examination of the AEA clarifies and evaluates a common objection to enhancement, while providing a lens through which to analyse accounts of the nature and the value of achievement.

## Articulating the argument against enhancement from achievement

The AEA is not explicitly formulated but rather gestured at by both its critics and proponents. A version appears in Robert Nozick’s *Anarchy, State, and Utopia.* In his discussion of hedonism, Nozick introduces a “transformation machine,” a machine that could “transform us into whatever sort of person we’d like to be” (1974, 44). He remarks that one would not “use the transformation machine to become as one would wish” (44). Indeed, some would be reluctant to use the machine “at all” (44n). Nozick also introduces the “experience machine,” a simulation machine equipped to supply any pleasurable experience one desires. Nozick imagines that the experience machine offers on balance more pleasure than one can receive in the world outside the machine. Still, he thinks that, as with the transformation machine, we would be reluctant to hook up to the experience machine. This suggests that things other than pleasurable experiences matter to us, including being a certain way (43, 44). Our reluctance to use the transformation machine, he argues, indicates that “something matters in addition to one’s experiences *and* what one is like” (44; italics in original). Nozick suggests that we would not use the transformation machine because it would mean accomplishing things “easily”; there would be, if the machine was used indefinitely, “no limits we *need* to strain against or try to transcend” (44n; italics in original). The problem with the transformation machine, then, appears to be that it equips one with capacities that make it easy to accomplish things that are ordinarily difficult. What is lost is something like achievement—one actively bringing about some end through a challenging process.

Another instance of the AEA appears in Michael Sandel. He claims (2007, 25) that “[i]t is one thing to hit seventy home runs as a result of disciplined training and effort, and something else, something less, to hit them with the help of steroids or genetically enhanced muscles. Of course, the roles of effort and enhancement will be a matter of degree. But as the role of the enhancement increases, our admiration for the achievement fades. Or rather, our admiration for the achievement shifts from the player to his pharmacist.” On his view, “[t]he more the athlete relies on drugs or genetic fixes, the less his performance represents his achievement” (26). Similarly, Eric Juengst asks whether achievements realized with the aid of enhancement might be “hollow” and so lacking the noninstrumental value they would ordinarily possess (1998, 39).

A more precise AEA may be expressed as follows.

*The Argument against Enhancement from Achievement:* Enhancement undermines achievement either by interfering with something qualifying as an achievement or by interfering with achievement’s nonderivative value.

To specify the AEA further, something must be said about the nature and value of achievement. There is consensus that an achievement comprises a product and a process (Bradford 2015, 12ff.; Hirji 2019, 526–27, 538, 543; Hurka 2011, 97–118; Portmore 2007, 1–2). These can be distinct, as in performing research on a disease and discovering a cure, or the same, as in running a marathon. On the most developed accounts, an activity counts as an achievement just in case (i) the process is difficult and (ii) the agent competently causes the product (Bradford 2015, 25; von Kriegstein 2019a, 60; Hurka 2006, 221; 2011, 106, 98). When an activity is not difficult and/or not competently caused, it fails to qualify as an achievement.

Broadly, there are two views on how achievements acquire value: perfectionist and welfarist. On the former, achievements are valuable because they involve perfecting certain of our capacities (Bradford 2015, 121–23; Hirji 2019, 541–42). According to the most prominent perfectionist view, the capacities perfected or excellently exercised in achievements are rationality and willing (Bradford 2015, 121–23; for other varieties of perfectionism, see Hirji 2019; Hurka 2006). On welfarist views, achievements are valuable because they are noninstrumentally good for or beneficial to welfare subjects. Achievements might be good for one because engagement with reality makes one noninstrumentally better off, and achievements in part involve agents conforming reality to their intentions, or because meeting one’s goals makes one noninstrumentally better off and achievements are a subset of one’s goals (Keller 2004), or because achievements themselves make one noninstrumentally better off (Griffin 1986).

On the first version of the AEA, enhancement causes an activity not to involve the requisite amount of difficulty. On the second version, enhancement interferes with the agent causing the product in the right way. In each case, enhancement prevents an activity from qualifying as an achievement. On the third version, what one achieves with the aid of enhancement is, because of what is involved in the achievement, lacking in the properties providing its value.

In addition, it seems that each of the three versions of the AEA—focusing on difficulty, causation, and value—can be given a strong or a weak reading. On the strong reading:

*The Strong Argument against Enhancement from Achievement:* Enhancement undermines achievement either by preventing something from being an achievement or by emptying it of its nonderivative value.

At least some AEA proponents seem to hold this version. However, it might be implausible that enhancement either altogether prevents activities in which it is used from qualifying as achievements, or that it completely eliminates their nonderivative value. We might therefore also consider an alternative, weaker, reading of the AEA:

*The Weak Argument against Enhancement from Achievement:* Enhancement undermines achievement either by making something a lesser achievement or by reducing its nonderivative value.

It might be more charitable to attribute this, weaker, reading of the AEA to its proponents. On this view, the concern is that enhancement would, rather than preventing some activity in which it is used from being an achievement, render it a lesser achievement, or, rather than eliminating an activity’s nonderivative achievement value, reduce it.

To give the AEA the fairest possible hearing, we consider both the strong and the weak readings of the argument’s various renditions.

## Enhancement and difficulty

On the strong reading of the AEA’s first version, which focuses on difficulty, enhancement makes activities that would otherwise have constituted achievements “too easy,” thereby preventing them from being achievements.^2^ Call this:

*The Insufficient Difficulty Argument:* Enhancement undermines achievement by reducing the amount of difficulty involved in realizing the product below that which is required for it to qualify as an achievement.

Many hold the intuitively plausible view that difficulty is an important feature of achievements and that it involves effort.^3^ The thought is that provided one exerts a sufficient amount of effort, one’s activity counts as difficult and therefore as an achievement. One natural idea is that the amount of effort involved in an activity is determined by the intensity and duration of the effort exerted. The greater the sum of effort, the more difficult the activity.^4^

If enhancement made activities effortless, as suggested by the strong reading, it would prevent them from qualifying as achievements, for such activities would not be difficult. Consider Gwen Bradford’s (2015, 31) example:

*Virtuoso:* Heifetz, the great violin virtuoso, effortlessly tosses off a flawless performance of the complex Paganini caprices.

Playing the Paganini caprices is famously difficult, Bradford observes, but it is not difficult *for* Heifetz (2015, 38). Since playing the Paganini caprices is not difficult for him, it cannot be an achievement for him. Enhancement might have similar effects. Consider:

*Enhanced Violinist:* Nigel is a violinist for whom playing the Paganini caprices would ordinarily require sufficient effort for it to count as difficult. For Nigel, then, playing them would ordinarily be an achievement. One day, Nigel takes an enhancement which enables him to play the Paganini caprices as well and as effortlessly as Heifetz.

It seems that in this case, the lack of effort on Nigel’s part, like the lack of effort on Heifetz’s*,* prevents the playing of the Paganini caprices from being difficult for him and hence an achievement for Nigel. If all cases of “achievements” involving enhancement looked like *Enhanced Violinist*, then, we might have to grant that enhancement undermines achievement.

But it seems unlikely that all enhancement would involve enabling agents to accomplish things *without* effort, or with very little effort. Consider:

*Distracted Susan:* Susan is studying to become a physicist. Her studies are demanding, and although she is very intelligent and academically accomplished, she needs to employ a variety of strategies to succeed, including studying hard for exams, reading up on effective revision and time management strategies, and so on. In trying to complete some assignments, Susan finds herself easily distracted. Her mind wanders off, she must reread passages, and so forth. She decides to take a drug that some of her classmates use as a “cognitive enhancer,” which is said to improve concentration. Susan takes the drug; she finds that it helps her concentrate. She continues to study very hard, employing her revision and time management strategies. She eventually succeeds in becoming a physicist.

In this case, Susan’s success does seem to involve a sufficient amount of effort for it to count as difficult for her, even while enhanced. It is not clear, therefore, that enhancement vanquishes her achievement. Of course, had Susan’s success been accomplished *sans* enhancement it might well have been a *greater* achievement. This is consistent with our claim that the use of enhancement does not necessarily entail that some process-product amalgam fails to qualify as an achievement. Just like it is possible for A to exert more effort than B, but for neither A nor B to exert the requisite amount of effort for their activities to count as achievements, it is possible for C to exert more effort than D, but for both C and D to achieve something.

The AEA proponent might reply that we have not specified exactly the amount of effort required to qualify something as an achievement.^5^ So it is unclear that what Susan does qualifies as one. The problem with this reply is that the AEA does not itself specify how much effort is required to make something an achievement. Without an account of precisely how much effort is sufficient for difficulty and of how enhancement prevents users from exerting it, the reply will not work. Moreover, whatever quantity of effort the AEA relies on, it is unlikely to perfectly track the enhanced/unenhanced distinction, such that *all* enhanced activities will lack the requisite amount (and thus fail to qualify as achievements), and *all* unenhanced activities will possess it (and thus qualify as achievements).

We noted above that in accomplishing her goal without enhancement Susan’s achievement might have been greater. The AEA proponent may argue that this involves conceding the weaker reading of the AEA on which all enhanced activities involve *less* effort, compared to those undertaken without enhancement. They are therefore, other things being equal, lesser achievements than their unenhanced counterparts.

This is not a plausible strategy for the AEA proponent, for two reasons. First, it puts the proponent of the AEA in the position of having to explain why we should be worried in particular about the impact of *enhancement* on achievement. AEA proponents hold that enhancement poses an unusual challenge to achievements.^6^ But there are a great many things that make activities less effortful, including equipment (running shoes), trainers or tutors (in sport and in education), and natural endowment. The weak AEA might be able to establish that enhancement in some way diminishes effort, but it cannot yet establish that enhancement is any more problematic than other things that also decrease effort.

Second, at most the weaker version of the AEA establishes that enhancement may make some of the activities that we now think qualify as achievements lesser achievements. But it is certainly possible to imagine other activities that would be achievements *for* enhanced agents and which would be just as much achievements *for them* as certain activities are for unenhanced agents. What counts as an achievement might change in some cases, but there would remain plenty of activities that count as solid achievements.

The AEA proponent might try to salvage her claim that enhancement undermines achievement through eroding difficulty by abandoning the idea that difficulty requires effort. Not everyone agrees that difficulty is always to be analysed in terms of effort. Thomas Hurka holds that an activity’s difficulty depends in some cases on “how much skill and ingenuity … [it] requires” (Hurka 2006, 221). Hasko von Kriegstein (2019a, 55ff.) develops this idea arguing that though some activities do not require effort, they are nonetheless difficult because they are activities at which most adult human beings with average capabilities are likely to fail. While effortless, what Heifetz does is still difficult and hence an achievement because there is a low probability that those with average capabilities will succeed at it. Heifetz’s achievement is a matter of skill and talent, not effort. Let’s grant this. This does not clearly help the advocate of the AEA, for (a) they emphasise effort in their arguments and (b) one can be enhanced and employ talents and skills.

Von Kriegstein defends an agent-neutral conception of difficulty (2019a, 56). On the agent-relative account, an activity’s difficulty is a matter of how difficult it is *for* a particular agent. This conception of difficulty fits nicely with the idea that an activity’s difficulty is a function of how much effort it requires from a particular agent. On the agent-neutral account, an activity’s difficulty is a matter of how difficult it is for any “adult human being with average capabilities” to succeed at it (2019a, 61). It is quite possible that on the agent-neutral conception of difficulty enhancement facilitates achievement by making it the case that agents can do things (perhaps using talents and skills) at which most agents with average capabilities would be highly likely to fail.

Furthermore, it is unlikely that, by adopting some other conception of achievement, the AEA proponent can save her claim that enhancement threatens achievement by diminishing effort. Consider Sukaina Hirji’s account on which “an achievement is a process culminating in a product that is competently caused and that *tests the limit of an agent’s perfectionist capacity*” (2019, 543; italics in original). Perfectionist capacities include “one’s practical rationality, one’s theoretical rationality, one’s creativity, and one’s physical abilities” (542). Hirji holds that it is possible for one to achieve something in sport or in art simply through the (effortless expression) of talent or skill. She thinks that Heifitz’s effortless playing of the Paganini caprices is an achievement; he competently causes the playing and it tests the limit of his creative (perfectionist) capacity (537–38). Because Hirji’s view denies that effort (and also difficulty) is a component of achievement, AEA proponents cannot rely on her view to capture the threat that diminished effort might pose to it.

On the contrary, Hirji’s view makes achievements for enhanced individuals possible because such individuals can engage in activities that (seemingly) competently cause products and that test the limit of their perfectionist capacities. Suppose that Nigel is extraordinarily talented and skilled at playing the violin—he is gifted—but he is easily distracted (lacking focus and motivation). Suppose further that he takes an enhancement that increases his motivation and his focus just enough that he competently causes his playing the Paganini caprices effortlessly in accordance with his raw talent and skill. On Hirji’s view, this counts as an achievement. In this case, not only does enhancement not interfere with achievement, it facilitates it. This ought to make those who worry that enhancement interferes with the “cultivation and display of natural talents” (Sandel 2007, 29) less chary of them.

To defend the claim that enhancement undermines achievement, then, the AEA proponent will need to rely on another version of their argument. Although there is no consensus that effort and difficulty are essential to achievement, there is agreement that among achievement’s essential features is competent causation (Bradford 2015; Hirji 2019, 527, 528, 543; Hurka 2011; von Kriegstein 2019a; 2019b). The proponent of the AEA might consequently be on stronger ground arguing that the reason that enhancement threatens achievement is that it undermines competent causation. It is to this version of the argument that we now turn.

## Enhancement and competent causation

On the second version of the AEA, enhancement reduces the agent’s causal contribution to an achievement, making it questionable whether the achievement counts as hers.^7^ Call this:

*The Errant Causation Argument*: Enhancement undermines achievement by interfering with the agent playing the right causal role in a given activity, thereby reducing the extent to which the achievement is attributable to the agent.

Many hold that, in addition to the agent exerting sufficient effort or engaging in a difficult activity, a causal connection of the right kind between the agent and what she produces must exist in order for it to be *her* achievement.

There is agreement that this connection is forged by an agent’s intention and her action on it. The agent must intend and act to produce the outcome. This idea is variously expressed. Hurka (2011, 98) insists that for something to count as an achievement one has to “intend to produce some goal and then do so.” T. M. Scanlon (1998, 121) maintains that for the fulfilment of a rational aim to count as a success it must be “acted upon” and be “given a role in shaping … [one’s] other activities and plans.” Douglas W. Portmore (2007, 3) says that “achieving a goal involves having that goal realized due in part, at least, to one’s own efforts… . To achieve one’s goals, one’s efforts must be productive—that is, efficacious in bringing about their intended effect” (see also Keller 2004, 36). This suggests that one’s intentional pursuit of a goal must involve some plan of action that will (or is likely to) produce that goal. As Hurka (2011, 98) puts it, “achieving a goal can’t be a matter of luck. You have to have pursued the goal intelligently, or in a way that, given what you knew beforehand, made your succeeding likely.”^8^

Bradford provides the most robust account of the (intuitively plausible) requirement that for there to be an achievement there must exist a connection (of the right kind) between the agent’s actions and what she produces. In this way, Bradford distinguishes what is attributable to the agent from what is a matter of luck or similar factor (2015, 13–17). According to Bradford, in order for the product of an agent’s activities to count as an achievement attributable to her, it must be the result of (intense) effort that she exerts and be *competently caused* by her. Competent causation is analysed by Bradford as follows:

*A* competently causes *E* via φ-ing when *A*’s φ-ing causes *E*, and *A* at some point while φ-ing has the requisite amount of ungettierized justified true beliefs (JTBs) about his φ-ing causing *E.*

(72, 71, 73, 79)

The idea, then, is that one competently causes an outcome when one has the requisite amount of JTBs about one’s activity causing the product while one is acting to cause it (64, 73, 74). Some beliefs—general, structural beliefs—are weighted more heavily than mere discreet beliefs (68).

Might enhancement prevent the competent causation condition from being met so that an agent’s product is not her achievement? It may if an enhanced agent causes the product without holding the requisite amount of JTBs about the role enhancement played in realizing the product. Consider:

*Unwitting Effective Altruism*: Larry is aiming for effective philanthropy, but has a propensity to give to ineffective, so-called “warm glow” charities. Peter puts a moral enhancement in Larry’s tea. The moral enhancement aids Larry in avoiding giving to “warm glow” charities and promotes his giving to the most effective charities as determined by GiveWell. Larry is, however, unaware of having ingested the moral enhancement and about its effects on him.

Let us assume that, for Larry, given his propensity for warm-glow giving, avoiding ineffective charities involves the amount of effort (or difficulty) required for achievement. Would the moral enhancement, then, prevent what would otherwise have been an achievement from being so? If the enhancement is highly effective, Larry will refrain from committing the error of warm-glow giving, but he will not hold a sufficient amount of JTBs about the (very significant) role that the enhancement plays in the product, nor will he hold any JTBs about what is moving him, Peter’s role, and so forth. Larry would in addition probably hold some false beliefs about what is moving him, the relationship between his intentions and his actions, and so on. We might think that in a case like this, therefore, the competent causation condition is not met.^9^

If all cases of so-called “achievement” involving enhancement looked like *Unwitting Effective Altruism*, enhancement would (it seems) threaten achievement by preventing the agent from competently causing the outcome. But again we might think it unlikely that enhancement would *always* prevent products from being competently caused. In respect of the competent causation condition, it is the agent’s (lack of ) JTBs about how the enhancement affects what the agent is doing that threatens achievement. Larry lacks JTBs about the relationship between what he intends and what he does. When enhancement is deliberately used by an agent to make an activity that remains difficult possible through mastery of the means to one’s ends, it would seem that the competent causation condition could be met. Consider:

*Moral Self-enhancement *: Bill is a fervent effective altruist. He currently donates a significant proportion of his income to highly effective charities. Bill wishes to boost his charitable giving, but finds it hard to do so. He has a sturdy will, but is hampered by some residual parochial beliefs about the limits of morality. He undergoes an operation to have a device implanted in his brain that helps him resist the influence of these beliefs on how he acts. Bill is aware of having undergone the operation and its effects, and thus holds a number of JTBs about the moral enhancement and its role in his actions to increase his effective altruism. Even when enhanced, Bill still needs to do various things to be more effective and to successfully resist the influence of parochial beliefs on his actions. Bill also holds a number of JTBs in respect of these additional activities and their role in producing what he intends. Being a more effective altruist, then, requires Bill to expend the amount of effort (suppose) required for achievement.

In this case, the difficulty condition is met. But it seems possible that the JTBs Bill holds about the moral enhancement and its relation to the outcome (his various activities and their effects, and so on) might be sufficient for the competent causation condition to be met, too. Bill causes the outcome via φ-ing (which includes his various activities and strategies, of which the moral enhancement is one), and he has the requisite amount of JTBs about his φ-ing (including his use of the moral enhancement) causing the outcome at some point while φ-ing. The moral enhancement does not, then, prevent Bill’s more effective altruism from being an achievement for him.

In reply, the AEA proponent could try to specify the number of JTBs that are required for competent causation so that enhanced agents are excluded from being competent causes. The precise amount of JTBs one needs to hold about a process and a product in order to competently cause that product is not fixed. The AEA proponent might argue that Bill does not hold a sufficient amount of JTBs to be competently causing the outcome because he does not, for example, hold a sufficient number of beliefs about the mechanism by which the moral enhancement works. Therefore, the AEA proponent might contend, Bill does not hold a sufficient amount of JTBs about how he produces his intended outcome via the moral enhancement (and his other actions).

This move is unhelpful to the AEA proponent. It is not clear that any threshold he sets for the amount of JTBs required will exclude all enhanced activities from being competently caused. Moreover, it is likely that any threshold that is set high enough to exclude enhanced agents from competently causing an outcome would also exclude a great many activities that proponents of the AEA accept as achievements, including activities that are produced in part through (the gift of ) raw talent and acquired skills. Since the AEA proponent needs to exclude enhanced agents from being competent causes for his argument to succeed, it seems that at least the strong reading of the *Errant Causation Argument* fails.

The AEA proponent might respond that he did not intend the strong reading of the AEA according to which all enhanced activities are precluded from being competently caused, but rather the weaker reading which holds that achievements by agents who are enhanced are lesser achievements because they are less competently caused. After all, some individuals who take an enhancement might have only a few JTBs about its role in the outcome. This decreases to some extent the agent’s causation of the outcome.

However, this is a poor strategy for the AEA proponent. As noted, the proponent thinks that *enhancement* poses a *special* threat to achievement. That is, he appears to hold that it is unlike other tools an agent might deliberately employ to realize intentions. Other tools, such as sophisticated equipment and training, might also undermine an agent competently causing an outcome if she is unaware of the role it plays in her success. The AEA proponent either must concede this, and then abandon thinking that enhancement poses a special threat, or he must provide an account of how these other things differ from enhancement such that they do not (like enhancement) undermine the agent’s competent causation. In the absence of such an account, the weak reading of the AEA will not successfully establish that enhancement poses a general and unusual threat to achievement.^10^

One might suggest here that by adopting some other conception of competent causation, the AEA proponent could save the claim that enhancement undermines achievement. One of the main rivals to Bradford’s account holds that an agent competently causes some outcome when she (a) takes actions that increase the likelihood of the outcome and (b) performs the actions because they increase the likelihood of the outcome (von Kriegstein 2019b).

This conception will not save the claim, for it is not clear that on this view the mere fact that an agent is enhanced is sufficient to disrupt her action competently causing an outcome. Consider again *Moral Self-enhancement.* Bill undergoes a surgical procedure that enhances him and while enhanced he acts to increase his effective altruism. His undergoing the enhancement increases the likelihood of the outcome and he undergoes the enhancement precisely because it will increase the likelihood of his actions producing his intended outcome. On this view, then, it is possible to be enhanced and competently cause an outcome.

The AEA proponent might urge that the conceptions of competent causation so far considered fail to properly capture how enhancement interferes with the agent playing the right causal role in bringing about their intended outcome. He might suggest that the focus should be less on taking actions that increase the likelihood of one’s intended outcomes or on having a sufficient number of JTBs about what one is doing while one is doing it and more on the role that the enhancement plays in producing the outcome. The view would be that to the extent to which one is enhanced one is less of a cause of an outcome, however knowledgeable or intelligent one may be in the pursuit of one’s outcome, such that the outcome is less attributable to one’s agency and more attributable to the enhancement.^11^

It is difficult to make sense of such a view. This is because it is difficult to comprehend how one might (a) know what one is doing while one is doing it, (b) cause an outcome, (c) exert a sufficient degree of effort (or skill or talent) towards it, and yet not have the cause attributed to one’s agency but rather to (at least in part) an enhancement. The burden should be on the proponent of the AEA to show in what way one’s agency is not the cause of the products of one’s actions when one knows what one is doing while doing it or when one takes actions that increase the likelihood of one’s intended effects. All the view says at present is *that* enhancements cause or serve as a greater cause of the products of those undergoing them; it does not state *how or in what way* this is the case.

A view on which it is possible for one to pursue one’s goal intelligently or knowledgably, be engaged in a difficult activity directed to some end, produce it, and yet not be the cause of it, is metaphysically obscure. The idea behind it seems to be that if an agent’s enhanced capacity is involved in bringing about some product, the capacity, not the agent, produces it. This involves separating an agent from her capacities. However, it appears hard to do so. We tend not to distinguish an agent from her raw talents or honed skills, so that the talent or skill, not the agent, is producing whatever they have a role in producing. So it is not clear how or why it would be done in the case of capacities.

Imagine that we could separate an agent from her capacities and her talents. This would not save the view that enhanced capacities are unique in interfering with agency. For the AEA proponent offering it will have to either concede that in cases in which raw talents or honed skills are at play the agent is also less of a cause of the outcome or explain how enhanced capacities are different such that they, but not talents and skills, erode agency or causation.^12^

There is another way in which enhancement might undermine an individual agent’s competent causation: by dispersing it across a group of agents. Imagine a case in which an individual is enhanced to have fins and flippers and a more aquadynamically shaped head in order to improve her swimming speed.^13^ Intense effort by the swimmer is required to maximize the fins and flippers in her swimming endeavours. The other agents who play a role in the design, development, and construction of the fins and flippers have also devoted considerable intense effort to their tasks. The swimmer surely has some JTBs about her new physique and its impact on her swimming. But she might well lack JTBs about the manufacturing, design, and exact powers of the fins and their interaction with her own capacities to produce her swimming speed. In particular, she might lack general structural beliefs unifying all these particular beliefs. The designers, developers, and fabricators might also have JTBs about their aspect of the project, but not about the swimmer’s exact interaction with the new technologies. In this case, it might not be clear that the agent’s swimming accomplishments are competently caused by her or anyone else and so they would fail to be achievements *for her* or any other individual. But while we might not have an achievement *for the individual*, the new swimming speed is an achievement *for the group*: the swimmer, developers, designers, and so on, who together possesses the requisite amount of JTBs to competently cause the outcome.

Enhancement might be more likely to make an activity a group achievement rather than an individual one, since an enhancement will, in most cases, have been developed by someone other than the agent. The agent may as a consequence lack the requisite number of JTBs about how the enhancement works. But group achievements will not be unique to enhanced activities. The most obvious example is team sports in which no one individual alone competently causes an outcome that counts as an achievement (such as winning a championship). However, group achievements are also common in sports in which an individual's success depends on coaches, nutritionists, and those who design the equipment used in the same way as in the swimming example above. If the proponent of the AEA allows for group achievements in such cases, it will be hard to deny them in cases of enhancement.

In reply, the proponent of the AEA could deny that group achievements are real achievements. This seems a rather large bullet to bite. But if the proponent allows group achievements in, for example, team sports, she will need an account of competent causation that explains that whereas group accomplishments of this sort are competently caused, enhanced group accomplishments are not.^14^

If our foregoing arguments are right, it is possible for the activities of enhanced agents to count as achievements.^15^ But it does not follow that such achievements have value. The AEA proponent may rely on a different version of the argument, which says that while it may be true that an enhanced agent can achieve something, such achievements are valueless. It is to this version of the AEA that we now turn.

## Enhancement and the value of achievement

In the previous sections, our focus has been versions of the AEA on which enhancement undermines achievement either by preventing it from being difficult or competently caused. The third version of the AEA maintains that achievements brought about with the help of enhancement are hollow or lacking in value.^16^ On the strong reading of this argument, the AEA holds that enhancement makes an achievement valueless. On the weak reading, it holds that enhancement renders an achievement considerably less valuable than it would be *sans* enhancement. Call this:

*The Value-Reduction Argument*: Enhancement undermines the value of achievement by interfering with its sources.

To make sense of this, we turn to accounts of the value of achievement. Bradford has the most developed account. She holds that difficulty and competent causation are directly responsible for achievement’s noninstrumental value (2015, 114),^17^ and that the value of difficulty and competent causation is perfectionist in nature (114–123). Perfectionism is the view that noninstrumental value is located in the excellent exercise or perfection of certain capacities that are characteristic of human beings which, on Bradford’s view, comprise the capacities of rationality (theoretical and practical) and willing (118–19). Difficulty and competent causation involve the exercise of the capacities of rationality and willing, therefore they possess noninstrumental value (121–123). Because achievements in turn involve difficulty and competent causation achievements are noninstrumentally valuable.

If AEA proponents contend that enhanced achievements are “hollow” or “cheap” or lacking in value, they cannot subscribe to Bradford’s view. For her, achievements are *always* valuable because the essential features of achievement are directly responsible for their value. Any and every achievement possesses value because any and every case of achievement involves the exercise of the two characteristic capacities of rationality and willing that confer value on difficult activity and on competent causation. Insofar as AEA proponents accept this variant of perfectionism, they cannot hold that there are hollow achievements. This is an unfortunate outcome for AEA proponents since something like Bradford’s view, which emphasises the will and effort, seems to be presupposed by their line of argument.

Not only does reliance on something like Bradford’s view fail to provide for the possibility of hollow achievements, it leaves the AEA proponent open to an objection. On Bradford’s view, all exertions of effort are valuable. It would seem to follow that “we should make every task that we undertake as difficult for ourselves as we can” (93).^18^ Proponents of the AEA seem to agree that exertions of effort supply value to achievements. They admire “those who overcome obstacles and struggle” (Kass 2003, 21) and who “sweat and struggle, sometimes against great odds” (Cole-Turner 1998, 155). If we should not enhance ourselves to make things less effortful because this alters the value of our achievements, why should we not make things *more* effortful to get more (achievement) value?

Bradford replies that effort is only one value among many (2015, 93). True, the argument goes, the fact that something is effortful provides *a reason* to value it. But it does not follow that making every activity more effortful is what we have, *all things considered*, reason to do, for there are things other than effort that are noninstrumentally valuable and that in certain cases outweigh the value of effort, for example, pleasure and knowledge (93, 134n2). The perfectionist AEA proponent may borrow this reply.

This will not help; the reply accepts that effort always has noninstrumental value. But it seems that not all effort possesses noninstrumental value. Therefore, not every case in which effort is eschewed is one in which it is outweighed by a rival value. Consider:

*Tony’s Headache*: Tony has a headache. This makes working on his paper more difficult, because he has to exert intense effort to concentrate. He can, however, take a pill to get rid of the headache and make work less effortful for him.

It is not obvious that the effort generated by the pain gives Tony a reason to refrain from taking the pill. And if he does take it, it is unlikely to be because the value of effort is outweighed by another value (for example pleasure or knowledge). Tony does not need to consult other values to provide a reason to take the remedy. The best explanation for this is that the exertion of effort is not in every case valuable. Since (at least) this perfectionist version of the AEA accepts that the exertion of effort is a source of value in achievement, *Tony’s Headache* presents a challenge to it.

Bradford might reply that in this case there is reason not to promote the thing that generates the need for effort, namely, the pain.^19^ Her view is that activities are effortful because of *effort-requiring features* (2015, 105–7). A wide range of features make activities effortful (for there are not, on her view, any features that are universally effort-requiring). Thus a math problem is difficult in virtue of complicated steps and skills, running a marathon is difficult in virtue of physical exertion and fatigue, and so on. The effort-requiring features, on Bradford’s view, are distinct from the effort itself. In *Tony’s Headache*, among the effort-requiring features is *pain.* But perhaps unlike complexity and physical exertion the effort-requiring feature of pain is something that we lack a reason to promote. Some effort-requiring features are ones we have reason to promote and others not, and which category a feature falls into depends (one might suppose) on what is of value.^20^

The AEA proponent may try to borrow this reply and further point out that *Tony’s Headache* is a case of treatment, not enhancement. The effort-requiring features in treatment cases (pain, illness, disability, and so forth) are of a kind that we do not have reason to promote. If *Distracted Susan* had suffered from attention-deficit hyperactivity disorder, we would have reason to reduce her effort by means of a concentration-promoting medication, since the source of the effort would be a medical condition which, presumably, for the AEA proponent, would be an effort-requiring feature we have reason not to promote.

But it does not seem likely that it is only in treatment cases that the effort-requiring features are ones we have a reason not to promote. It is not clear that, if Susan were instead distracted, for example, because she was heartbroken after being left by her partner this would be an effort-requiring feature that we would have reason to promote (or not reduce). It seems unlikely that the distinction between those effort-requiring features that are worth promoting and those that are not would track the treatment-enhancement distinction such that we would have reason to promote effort-requiring features targeted by enhancement, but not have reason to promote effort-requiring features targeted by treatment. If it did not, the AEA would not be able to rely on Bradford’s reply to cases like *Tony’s Headache* to impugn enhancement cases.^21^

May the AEA proponent do better by adopting an account of perfectionism that avoids the controversy surrounding the claim that effort always has noninstrumental value?^22^ Consider again Hirji’s view according to which “an achievement is a process culminating in a product that is competently caused and that tests the limit of an agent’s perfectionist capacity” (2019, 543; italics removed). Her view seems to avoid the objection pressed against Bradford above by dropping emphasis on the will. On Hirji’s view, the capacities to be perfected are “one’s practical rationality, one’s theoretical rationality, one’s creativity, and one’s physical abilities” (542). Although effort might be something that is involved in perfecting these capacities, it is not something that, on Hirji’s view, we have direct reason to promote. Exercise of will is not valuable per se. According to Hirji, if something counts as an achievement, it will have (perfectionist) value. So, again, there are no hollow achievements. The AEA proponent cannot appeal to this view for help in demonstrating that there are hollow or valueless achievements.

It is likely that any plausible perfectionist view will include capacities that are intimately connected with at least some of the essential features of achievement. Most perfectionist views will be committed to the claim that it is noninstrumentally valuable to realize to a high degree our intellectual or cognitive capacities and (perhaps) something like our willing or our capacity to be “at work in the world” (see Brink 1989, 232ff.; Hurka 1993, 2011, 99–118), and these seem to be just the sorts of things that are involved in achievement. All the views on the nature of achievement discussed above hold that competent causation is essential. This involves in some way the exercise of the capacities of practical and theoretical rationality. As long as this is the case, achievement cannot fail to have value from the perfectionist point of view.

The proponent of the AEA cannot secure the existence of hollow or valueless achievements by adopting a welfarist view of their value. On some welfarist views, for example, the achievement of any goal has noninstrumental prudential value. According to Keller’s (2004, 36) version of this position, “[w]henever an individual achieves a goal, she is better off in one respect.” Moreover, on his view, the value of an achievement lies partly in the fact that it involves effort: “The greater the quantity of productive effort that an individual successfully devotes to the achievement of a particular goal, the more the achievement contributes to her welfare” (36). On this view, if something counts as an achievement, whether enhanced or not, it has (prudential) value. Therefore, adopting a welfarist view of this kind will not help the AEA proponent explicate the idea that there are hollow achievements, and it cannot explain why in some cases there is no reason to make activities more effortful. Indeed, insofar as a proponent of the AEA accepts *any* account of the value of achievement grounded in its essential features, they cannot argue that enhanced achievements have *no* value.

Other welfarist views are no friendlier to the idea that there are hollow achievements. As noted, James Griffin (1986) argues that accomplishments (his word for achievements) themselves are prudentially good for one. Therefore, in any case in which one achieves something one is noninstrumentally better off. On Portmore’s welfarist view (2007), the value of an achievement depends on the investment made in it, where this is a measure of the amount that an individual has personally sacrificed, for example, leisure, family time, and education, to achieve something. The more one has invested in the achievement, the more prudential value the achievement has since it redeems the sacrifice. This view does not rest the value of an achievement in its essential features. However, it is quite possible that enhanced individuals have sacrificed a lot personally for their achievements. In this case, an activity will have prudential value if it qualifies as an achievement. On Portmore’s view, then, not all enhanced achievements will be hollow.

On other welfarist views, some achievements may be hollow or cheap. Hedonist and (some) preference fulfilment views seem to allow for this. An enhanced achievement might be such that it fails to produce pleasure or satisfy one’s considered preferences. But this will not be true of all enhanced achievements, for some will produce pleasure or produce (considered) preference fulfilment or both, and it may be true that some nonenhanced achievements will be hollow. Hollowness is not, then, unique to enhanced achievements.

An objective-list view comprising such noninstrumental prudential goods as the contemplation of beauty, friendship, pleasure, autonomy, and so on might allow for hollow achievements. It is possible that if an achievement fails to produce any of these values, it is hollow. But again the view does not seem to generate the conclusion that all enhanced achievements lack value or are hollow. If an enhanced achievement advanced various goods such as friendship or autonomy, it would have value.

It does seem that if enhanced achievements make people’s lives go prudentially better in some way, and there is at least some reason to promote welfare, there is at least some reason to value some (safe and effective) enhancement. The move from perfectionism to welfarism does not, then, help the AEA proponent establish that enhanced achievements are hollow or lack value. The strong reading of this version of the AEA fails on both perfectionism and welfarism.

Moreover, we might think that there could be cases in which enhancement *increases* the value of achievements. First, with respect to effort, enhancing our capacities to exert more effort might result in an ability to do more difficult things yielding potentially more valuable products.^23^ Second, enhancing our capacity for rationality would increase our ability to understand and manipulate various elements of our activities and the process by which our achievements come about and, thereby, presumably increase our ability to successfully undertake even more complex achievements.^24^

What about the weaker reading of this version of the AEA? Its proponent could make the argument that rather than causing achievements to lack value, enhancement might cause them to have (considerably) less value.

This line of reply is not a good one for the AEA proponent for again it does not explain how enhancement poses an unusual threat to the source of the value of achievement. There will be all manner of interventions or tools that may make a difference to the source of the value of achievement, not all of which are so-called “enhancements.” The difficulty for the AEA proponent is that he has yet to supply a plausible way to distinguish the threat posed to the source of the value of achievements by enhancements from those posed by other interventions or tools so that only the former count as generally and unusually threatening to the value of achievement.

## Conclusion

At one time neglected, achievement is now a growing preoccupation in normative ethics. In this paper, we engaged with the nature and the value of achievement through a critical examination of an argument according to which biomedical enhancement of our capacities is impermissible because enhancing ourselves in this way would threaten our achievements. We called this the argument against enhancement from achievement, or the AEA. We clarified the argument to determine its strength and its effect on the philosophical understanding of achievement’s nature and its value.

We developed and assessed three versions of the AEA. On the first version of the argument, enhancement causes the activity not to involve the requisite amount of difficulty. On the second version, enhancement interferes with the agent causing the product in the right way. In both cases, enhancement interferes with an activity qualifying as an achievement. On the third version of the argument, what one achieves with the aid of enhancement is, because of what is involved in the achievement, lacking in the properties that typically provide its value. Each of these versions can be given a strong or a weak reading. We argued that strong readings of the AEA fail to establish that enhancement always interferes with the nature or value of achievement, and that weak readings, while in some cases successful in showing that enhancement interferes with the nature or value of achievement, fail to establish that enhancement poses an unusual threat to achievement.

## Footnotes

1
We take enhancement to be biomedical interventions used to “alter the characteristics of already healthy persons” (Douglas 2008, 228). For example, blood doping in cycling and Modafinil or Adderall use to improve focus or concentration. The AEA has primarily been raised in regard to enhancement of this sort, but it could also apply to nonbiomedical forms of enhancement (as could our arguments).

2
Sandel maintains that one, though not the main, problem with biomedical enhancements is that they “undermine effort and erode human agency” (2007, 26). The President’s Council on Bioethics says that “[i]n those areas of human life in which excellence has until now been achieved only by discipline and effort, the attainment of similar results by means of drugs, genetic engineering, or implanted devices looks to many people … to be ‘cheating’ or ‘cheap.’ Many people believe that each person should work hard for his achievements. Even if we prefer the grace of the natural athlete or the quickness of the natural mathematician—people whose performances deceptively appear to be effortless—we admire also those who overcome obstacles and struggle to try to achieve the excellence of the former. This matter of character—the merit of disciplined and dedicated striving—is surely pertinent. For character is not only the source of our deeds, but also their product” (2003, 291). The “heart of the matter,” they contend, is “understanding the true dignity of excellent human activity, and how some new ways of improving performance may distort or undermine it” (2003, 140).

3
Bradford (2015, 12, 26–63); Hurka (2006, 221; 2011, 106–16); Nozick (1974, 44); and von Kriegstein (2019a, 54–55) hold that achievements are difficult and analyze difficulty in terms of effort. Von Kriegstein and Hurka think that there are other conceptions of difficulty (discussed below). Keller (2004, 36) and Portmore (2007, 3) link achievement and effort.

4
Bradford argues that the account of difficulty that equates it with the sum total of effort exerted—the total view, as she calls it—has unintuitive implications. It entails what she calls the Ridiculous Conclusion: that for any activity of finite duration involving a very high degree of intense effort there is another activity involving very minimal effort but going on for a very long time that would involve on balance a greater sum total of effort and hence be more difficult (2015, 47). This is analogous to Parfit’s Repugnant Conclusion in population ethics (1984, 388). Bradford thinks this implausible. A very intense one-hour game of ice hockey is much more difficult than many hours of gently rolling a ball back and forth between one’s hands, even if the latter involves on balance much more effort. The Ridiculous Conclusion can be avoided by stipulating that only the sum total amount of effort beyond a certain critical level is counted as relevant to determining an activity’s difficulty. Bradford calls effort above such a critical level intense effort. Provided the sum total of intense effort is sufficient, the activity will include enough effort to count as difficult and hence an achievement (2015, 49–50). If one is convinced by Bradford’s argument, one can substitute intense effort for all uses of effort in the text.

5
Bradford concedes that setting this requires “further investigation” (2015, 56).

6
For the view that enhancement poses an unusual threat to achievement, see Cole-Turner (1998, 155); Juengst (1998, 39); Kass (2003, 21–23); and the President’s Council on Bioethics (2003). It is common to interpret proponents of the AEA as holding this view. Allen Buchanan (2011, 9) notes that authors such as Michael Sandel and Leon Kass (not just in their writing on enhancement and achievement, but in their writing on enhancement more generally) “try to discredit enhancement *in toto*, rather than just some enhancements under some circumstances”. Thomas Douglas (2019, 8) notes that even though “not all activities are such that their purpose is undermined when they are pursued with the aid of enhancements … some present the concern about devaluing achievements as a general worry about enhancement.”

7
For example, in its report on biomedical enhancement, the President’s Council on Bioethics seem to suggest that when enhancement is used, *it*, rather than the agent, might be causing the outcome: The runner on steroids … is still, of course, a human being who runs. But the doer of the deed is, arguably, less obviously himself and less obviously human than his unaltered counterpart … or more like the horses we breed for the racetrack than a self-willing, self-directing, human agent. He does the deed (running), and his resulting time may be measurably superior. But he is also (or increasingly) the passive recipient of outside agents that are at least partly responsible for his achievements (2003, 144). Elsewhere in their report, the President’s Council on Bioethics says that “it seems that some performance-enhancing agents … call into question the dignity of the performance of those who use them. The performance seems less real, less one’s own, less worthy of our admiration. Not only do such enhancing agents distort or damage other dimensions of human life … they also seem to distort the … activity itself … What is at stake here is the very meaning of human agency, the meaning of being *at-work* in the world.” (2003, 140–41). Sandel also worries that enhancement will “erode human agency” (2007, 26).

8
This is likely too strong. Luck will inevitably play a role in achievement, at least in most cases. For accessible discussions of this, see Pritchard 2010 and von Kriegstein 2019b.

9
We might worry that in such cases it is lack of effort that prevents the activity from qualifying as an achievement. It is hard to distinguish effort from causation in such cases because difficulty involves *directed effort* to some degree. We might think it unlikely that we would see the kind of directed effort required for the difficulty condition to be met in cases in which an agent is unwittingly enhanced. For the purposes of this section, we assume that the effort condition is met. This provides for a distinct version of the AEA which is not impugned by the previous section’s arguments.

10
It might be thought that the AEA proponent could deflect our response to the weak reading by jettisoning the claim that enhancement poses a special or unusual threat to achievement. But jettisoning this claim involves either admitting that one ought to be against things other than enhancement that interfere with one’s JTBs about one’s activities in similar ways and to similar extents (e.g., sophisticated treatment regimens) or giving up the argument altogether, for without the specialness claim there is nothing about enhancement in particular to be concerned with. Thanks to an anonymous referee for urging us to clarify this.

11
For an argument like this, see Kass 2003, 22–23.

12
An anonymous referee suggested that perhaps the difference between raw talents and honed skills, on the one hand, and enhanced capacities, on the other hand, is that while raw talents might be “constitutive” of the person and honed skills might be something for which she can take credit, enhanced capacities are not constitutive of the person and not something for which she can take credit. This suggestion is not very helpful. It is hard to know what does and does not qualify as constitutive of the person. To state that raw talents are constitutive is question begging. Furthermore, it is not clear that enhanced capacities cannot in part be something one can take credit for and so not completely unlike honed skills.

13
We owe this example to Gwen Bradford.

14
For a discussion of enhancement, ownership of achievements, and group achievements, see Goodman 2010, 155–57. An anonymous referee suggested that perhaps there is some loss when an activity counts as a group rather than an individual achievement. It is not obvious to us what that might be. It is not clear that it is better, say, to win an Olympic gold medal in snowboarding rather than in ice hockey (at least for the players who are not riding the bench). It is possible that something is lost, though it is also possible that something is gained that makes up for that which is lost.

15
We have conducted much of the discussion of competent causation in terms of JTBs. However, one need not rely on JTBs to interpret competent causation. Instead, one can rely on knowledge-how or understanding (Bradford 2015, 80–82). In this case, to competently cause some outcome one would have to possess the requisite amount of knowledge-how pertaining to or understanding of how one is causing some outcome while one is causing it. There has to be some such state—such as intelligent pursuit or intentional planning—playing a role in achievement. Whatever state one relies on, it is important that the requisite amount of JTBs or of knowledge-how or of understanding that one possesses is robust enough to explain how the agent is causally connected in the right way to what she achieves. The amount of understanding and so on required cannot be too great for achievements will then be too scarce; at the same time, the amount cannot be too miniscule for then achievements will be too common. It is likely that the requirement of having a sufficient quantity and quality of JTBs to competently cause an outcome is too epistemically demanding. Following Hurka (2011), you might think all that is required for competent causation is that the agent has beliefs about the rough probability of their desired outcome occurring given their intentional activity. But if we grant this, it is plausible that an enhanced agent can competently cause an outcome by possessing the right amount of beliefs about the likelihood that their actions (including being enhanced) will produce the desired outcome.

16
Kass (2003, 21) argues that “Yet in those areas of human life in which excellence has until now been achieved only by discipline and effort, the attainment of those achievements by means of drugs, genetic engineering, or implanted devices looks to be ‘cheating’ or ‘cheap.’ We believe—or until only yesterday believed—that people should work hard for their achievements. ‘Nothing good comes easily.’” Juengst (1998, 39) contends that enhancement interventions “short-circuit admirable human practices in an effort to obtain some personal goal.” They do not raise the question of “either causation or responsibility.” Rather, they raise the question of whether the accomplishment is earned; a goal accomplished in this way “is a hollow accomplishment, without the intrinsic value it would otherwise have.” Cole-Turner (1998, 155) says that “[t]here is … a glory or dignity in human accomplishment attained the ‘old fashioned way,’ through sweat and struggle, sometimes against great odds. It is something like the mountain climber who struggles to the top of a great peak, but then on flying home ascends quickly by jet over the top of the mountain, and looking down at the summit, feels cheated of the value of the original climb. Technology, precisely because of its power and efficiency, seems to cheat us of the experience of accomplishment, which is something valued in distinction from the achievement of the end.”

17
The process and the product might also add value to the state of affairs (Bradford 2015, 91, 99, 122, 133, 160ff.).

18
The same worry arises for Keller’s welfarist view which maintains that increased effort entails increased value (2004, 36, 38; also Hurka 2011, 99–118). For a criticism of Keller, see Portmore 2007, 9ff. For more on this, see below.

19
We owe this reply to Gwen Bradford.

20
Bradford might, of course, argue that there are all kinds of reasons against making activities more difficult. Additional effort leads to instrumental evils (exhaustion)—so we may have (extrinsically sourced) reasons to avoid difficulty, even though *pro tanto* difficulty generates reasons (and is valuable) for its promotion. But claiming that the reasons of effort are outweighed by instrumental reasons seems not to be a correct view of why we think Tony ought to avoid the pain. He has no reason to exert effort to deal with the pain even in the absence of instrumental disvalues. It seems likely that we value the exertion of great effort because it involves perseverance or tenacity and having the capacity to persevere in the face of great obstacles or challenges is of use in dealing with a great many things standing in the way of a good life, including headaches, inclement weather, and short-tempered co-authors!

21
Bradford’s reply seems to miss the point anyway. There does seem to be a reason, on her view, not to take the pill: not taking the pill makes the activity (writing the paper, in Tony’s case) more difficult. Qua painful there is a reason to take the pill; qua effort-requiring there is a reason not to take the pill. This seems odd. It seems that the fact that the effort is increased by the pain is not a reason to refrain from taking the pill.

22
Our intuition in *Tony’s Headache* that the exertion of effort is not always noninstrumentally good is controversial. We are aware that the case is divisive and not everyone shares our intuition; our aim is to bring out a potentially controversial element of one view of the value of achievement. Thanks to two anonymous referees for pressing us on this issue.

23
At least on some perfectionist views, a more valuable product means more perfectionist value attaches to the achievement (see Hirji, 2019, 535–36).

24
In reply to these claims, an anonymous referee puts forward a different argument against enhancement that the referee calls the average argument. On this argument, there is no achievement-based reason not to enhance, but nor is there an achievement-based reason to enhance. All enhancements are such that, while they may realize better products, their value as achievements remain the same, because the effort exerted in them is the same. To illustrate, the referee offers the following. Imagine that Fred is capable of running a seven-minute mile “using all” his effort. Fred takes an enhancement. He then runs a faster than seven-minute mile, again “using all” his effort. The referee argues that since in both cases all of Fred’s effort is being used the value of Fred’s achievement remains the same. If this is true of enhancement generally, the referee argues, there is no achievement-based reason to enhance. It is, however, questionable whether what counts as “all” Fred’s effort is the same in quantity in both cases. What reason might we have for thinking that it is? It is possible that, at least in some cases, enhancement might increase the amount of effort one might be able to exert. If so, there is an achievement-based reason to enhance. Of course, the referee’s argument works only if we accept that other things equal the value of an achievement increases as it becomes more effortful. We have given reasons against this view. But even if one accepts it, there is reason to be puzzled about the claim that the value of Fred’s achievement is the same in both cases. The less than seven-minute mile seems a greater achievement. The effort view cannot capture this, because it embraces an agent-relative conception of difficulty (which is analyzed in terms of effort). Perhaps in this case it is more plausible to adopt an agent-neutral view of difficulty (which is analyzed in terms of what an adult human being with average capabilities is unlikely to succeed at). But on this conception of difficulty, we may have achievement-based reasons to enhance since doing so makes it more likely that we will be able to do things that are agent-neutrally difficult.
